# Time of Flight Secondary Ion Mass Spectrometry for Characterization of Pt-Coated Porous Transport Layers in PEM Water Electrolyzers

**DOI:** 10.1021/acsanm.6c00919

**Published:** 2026-05-25

**Authors:** Genevieve Stelmacovich, J. David Arregui-Mena, Michael Walker, Jayson Foster, Samantha Ware, James L. Young, Guido Bender, Adam Paxson, David A. Cullen, Svitlana Pylypenko

**Affiliations:** † Department of Chemistry, 3557 Colorado School of Mines, Golden, Colorado 80401, United States; ‡ Materials Science and Technology Division, 6146 Oak Ridge National Laboratory, Oak Ridge, Tennessee 37831, United States; § Chemistry and Nanoscience Center, 53405 National Laboratory of the Rockies, 15013 Denver West Parkway, Golden, Colorado 80401, United States; ∥ 391481 Plug Power, Latham, New York 12110, United States; ⊥ Center for Nanophase Materials Sciences, Oak Ridge National Laboratory, Oak Ridge, Tennessee 37831, United States

**Keywords:** time-of-flight secondary ion mass spectrometry, porous transport layers, protective coatings, characterization, method development

## Abstract

Titanium-based porous transport layers (PTLs) and iridium-based catalyst layers (CLs) are two main components of proton exchange membrane water electrolyzers (PEMWEs). PTLs are typically coated with platinum to minimize interfacial losses and to support long-term operation. Optimizing coatings and the PTL-CL interface requires comprehensive characterization. This study establishes time-of-flight secondary ion mass spectrometry (ToF-SIMS) as a valuable technique for PTL characterization, addressing capabilities and limitations related to PTL morphology. A methodology was developed that uses a Cs^+^ sputter beam for dynamic depth profiling, with data collected in both positive-ion (MCs^+^) and negative-ion modes to generate depth profiles, 2D ion maps, and 3D ion reconstructions. ToF-SIMS detected relative differences in platinum-layer thickness between samples; these trends were validated by cross-sectional scanning transmission electron microscope (STEM) measurements and flat-titanium substrate controls. Interfacial oxide layers are identified in both ion modes, with enhanced oxide sensitivity in negative mode. The technique’s high sensitivity enables detection of nanometer-scale coatings and trace impurities within the bulk PTL structure. These results provide a methodological framework for analyzing Pt-coated PTLs, with the potential to extend to other components in PEMWEs and other electrolyzer systems.

## Graphical abstract

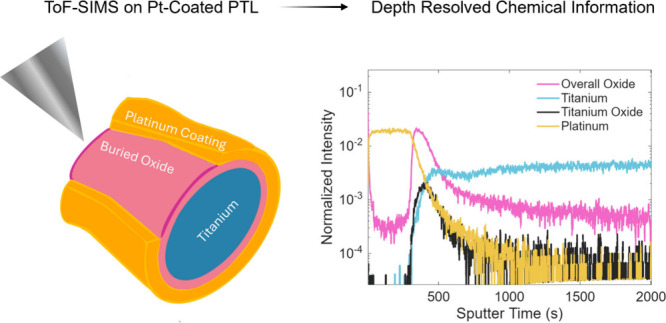

## 1 Introduction

Recent interest in the wide adoption of hydrogen energy has escalated the development of proton exchange membrane water electrolyzers (PEMWEs). PEMWE is a promising electrolysis system for scale-up approaches, as the compact design and durability of the PEMWE under operating conditions allow for high efficiency and durability in comparison to its competitors.^1,2^ Commercialization of the PEMWE drives the investigations toward reducing material and manufacturing costs.^3,4^

Titanium is the current state-of-the-art porous transport layer (PTL) material; however, there are still many concerns with its performance under operating conditions due to the naturally passivating titanium oxide.^5–9^ The added resistance of the oxide layer, specifically at the catalyst layer (CL) and PTL interface, leads to Ohmic losses that contribute to the overall overpotential of the cell. To mitigate these losses, PTLs are coated with a protective layer, which has been shown to significantly decrease interfacial contact resistance (ICR) and prevent titanium oxide growth during cell operation.^10^ Currently, platinum (Pt) is used as the state-of-the-art protective layer for the PTLs,^11,12^ but other platinum group metals (PGM) materials including Au have also been investigated.^13^ Significant research efforts have been dedicated to reduce the loading of PGM materials by creating thin PGM coatings, replacing PGM coatings with non-PGM alternatives, and through various post-treatments to modify the chemistry or morphology of the coatings.^14–24^ Detailed characterization is essential when optimizing and developing PTLs for maximum performance and long-term operation.

Various characterization methods, including scanning electron microscopy (SEM) and scanning transmission electron microscopy (STEM) paired with energy-dispersive X-ray spectroscopy (EDS), have been used to evaluate coated and uncoated PTLs, providing insights into morphology, elemental distribution in 2D, and coating layer thickness.^12,13,25^ However, EDS lacks chemical information necessary for assessing coating effectiveness and PTL degradation and thus has limited capabilities for understanding all layers and especially a buried oxide interface. Both SEM-EDS and STEM-EDS require sample preparation in order to evaluate cross sections of coated PTLs. Additionally, information on coating homogeneity across different sample areas is rather limited, especially in the case of STEM. X-ray diffraction (XRD), Auger electron spectroscopy (AES), and X-ray photoelectron spectroscopy (XPS) provide physicochemical information about the PTL and its protective coatings, but each is subject to distinct limitations.^17,21–23,26,27^ XRD is commonly employed to investigate phase composition; however, it is a bulk technique lacking spatial resolution. AES has been reported in conjunction with argon-ion sputtering for depth profiling, enabling *Z*-direction chemical analysis, but it is generally considered less chemically specific than XPS.^27^ XPS offers surface-sensitive chemical characterization but typically lacks spatial resolution on most instruments.^28,29^ Because of these individual limitations, multiple complementary techniques are typically employed to build a complete understanding of PTL and protective coating chemistry. Even so, spatially resolved chemical characterization in three dimensions remains a significant gap in the literature, particularly for buried interfaces in morphologically complex structures, such as PTLs.

ToF-SIMS, operated in dynamic sputtering mode, was first introduced for PTL characterization by investigating Pt, Au, and Ir protective coatings on titanium felt PTLs.^24^ In that study, ToF-SIMS was employed alongside STEM-EDS and XPS to investigate changes in protective coatings and the coating PTL interface before and after 4000 h of electrolysis under realistic operating conditions, tracking degradation across different PTL coatings. STEM-EDS was used to visualize the cross sections, demonstrating an oxide layer between the protective coating and the Ti baseline PTL. XPS was employed to identify oxidation states at both the surface and subsurface regions of the coated PTLs by incorporating argon sputtering for depth profiling. This approach enabled the tracking of metal-to-oxide ratios as a function of depth, corroborating STEM-EDS results while also providing additional insight into nature of oxides. Dynamic ToF-SIMS enabled three-dimensional, spatially resolved chemical mapping of these samples, demonstrating its potential for resolving layered structures and probing buried interfaces within PTLs.^30^ It identified key species associated with both the protective coatings and the underlying PTL, including titanium, titanium oxide, platinum, platinum oxide, iridium, and iridium oxide. Crucially, it also provided chemical insight into the buried interface between the protective coating and the titanium substrate, information not readily accessible using other techniques. Relative intensity changes observed before and after stability testing further demonstrated ToF-SIMS’s capability to track changes due to degradation processes. Collectively, these findings underscore ToF-SIMS’s potential to address persistent characterization gaps in PTL research.

Nonetheless, significant challenges must be addressed before ToF-SIMS can be widely adopted for the PTL characterization.^31^ The technique is best suited for relatively smooth or planar samples, as significant surface roughness or complex topography can cause local electric field variations and secondary ion signal attenuation (or ‘shadowing’), reducing spatial resolution, mass resolution, and ionization. As a result, ToF-SIMS has been more commonly applied to planar systems, such as semiconductor materials and thin films, where surface topography is more controlled. PTLs, by contrast, are highly porous and exhibit substantial surface roughness, which is further amplified by coating methods, such as physical vapor deposition and electrodeposition. These conditions can result in shadowing and variable sputtering rates, both of which reduce the spatial fidelity and complicate quantitative interpretation. Additionally, ToF-SIMS is considered quasi-quantitative; while it provides excellent chemical sensitivity, its ion yields depend on matrix effects and instrument conditions, making calibration and normalization essential for reliable comparisons.^32^ To ensure meaningful results, repeatable instrument parameters must be maintained, and optimally, calibration curves should be developed to accurately track coating thicknesses and chemical variations within PTLs.

This study explored ToF-SIMS capabilities and limitations to further establish it as a reliable technique for PTL characterization. A systematic evaluation was conducted across a series of representative coated PTL samples, focusing on challenges related to complex morphologies and buried interfaces. The investigation began with the analysis of Pt-coated felt PTLs with varying thicknesses of platinum deposited by sputtering. These samples had relatively low Pt content compared to standard Pt-coated PTLs, providing a good data set for method development for evaluation of the next generation of coatings. Initial evaluation demonstrated the effectiveness of ToF-SIMS in chemical identification by utilizing both positive and negative ToF-SIMS analysis modes. Pt-coating thickness differences were assessed by correlating ToF-SIMS depth profiling with STEM-EDS measurements as well as with a comparison of PTLs to flat Ti substrates which served as references. The ability to resolve buried oxide layers was assessed using both positive and negative ion modes, with negative mode providing enhanced sensitivity to oxide species such as TiO^–^ and PtO^–^. Further analysis of PTLs with thin platinum coatings evaluated the technique’s sensitivity to low-platinum loadings and contaminants. Three-dimensional chemical maps were reconstructed from ToF-SIMS data to visualize species distribution within sintered PTLs, demonstrating the technique’s potential for spatially resolved, volumetric analysis.

## 2 Experimental Section

### 2.1 Materials

A sample set of felt PTLs (2GDL-10-0.25, Bekaert)^34^ was coated with varying amounts of protective Pt coatings via sputter deposition to investigate low-loading protective coatings. This sample set consisted of PTLs with Pt deposition times of 1, 2.5, 5, and 10 min, along with PTL with no Pt coating serving as a reference. Flat titanium sheets (Grade 2 Ti sheet (McMaster, Ultra-Corrosion-Resistant grade 2 Titanium Sheet, 0.035″ Thick x 6″ Wide x 6″ Long, product number 9051K55)) were also coated in parallel, thus having the same deposition times. The PTLs were Pt coated on both sides by DC magnetron sputter deposition in a custom system using a 2″-diameter Pt target (99.99% ACI Alloys, Inc.).^35^ After loading the uncoated PTLs, the vacuum chamber was evacuated to at least 5 × 10^–5^ Torr before flowing Ar gas (99.999%) to establish a pressure of 10 mTorr throughout deposition. The supplied sputtering power was 20 W and the sample stage was stationary. Presputtering at the same conditions was conducted for 2 min before opening the shutter. Additional uncoated and Pt-coated sintered titanium powder and felt PTLs were provided by Plug Power.

**Figure 1:**
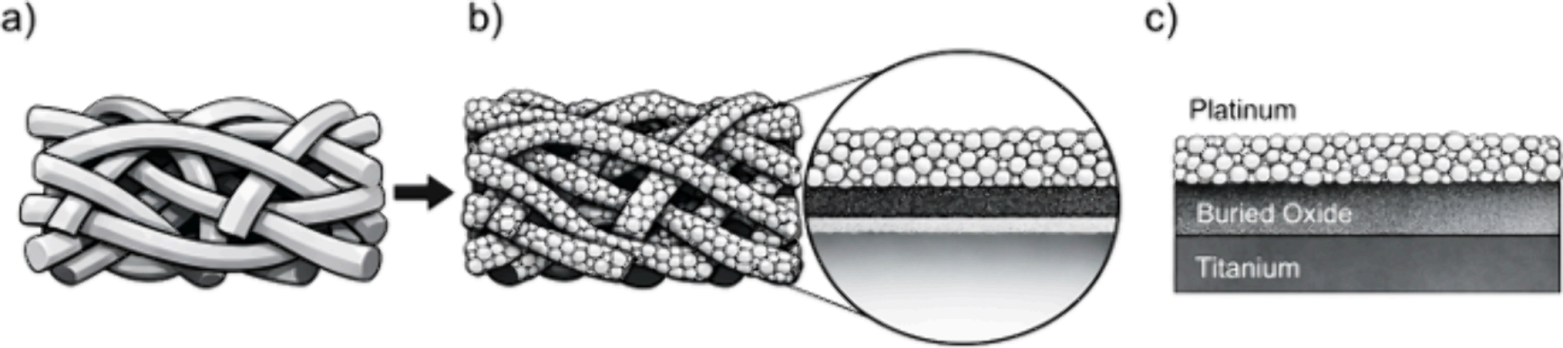
Cartoon schematic of Pt-coated PTL fabrication: (a) as-received titanium PTL, (b) Pt-coated PTL after sputter deposition, and (c) cross-sectional schematic of the Pt-coated PTL showing the platinum layer, buried oxide, and bulk titanium. This schematic was generated using artificial intelligence.

### 2.2 Instrumentation

#### Electron Microscopy

Lamella cross sections of the PTL coatings were prepared via the liftout method using a Thermo Fisher Scientific 3D Versa DualBeam FEG FIB-SEM. HAADF-STEM imaging and STEM-EDS analysis were performed in a probe-corrected JEOL JEM ARM “NEOARM” operated at 200 kV and equipped with dual silicon drift detectors with a total active area of 200 mm^2^. The EDS spectrum imaging and analysis utilized the JEOL Analysis Station software. SEM-EDS was conducted using the FEI Quanta 600I environmental SEM. Parameters for SEM imaging and EDS mapping include an accelerating voltage of 15 keV, a probe current of 10 nA, a working distance of 10 mm, and a magnification of 200x.

#### XPS

XPS was conducted using a custom Scienta-Omicron HiPP-3 system equipped with a R4000 hemispherical analyzer operating in swift acceleration mode and calibrated to the Au 4f region (83.95 eV) with a sputter-cleaned Au block. An Al Kα X-ray source (1486.6 eV) was used at 300 W and a 1.5 mm × 30 mm slit size and 50 eV pass energy to give a 187.5 meV approximate energy resolution of the analyzer. The analysis chamber pressure was maintained to be below 9.0 × 10^–8^ mbar and the analyzer pressure was below 5.0 × 10^–9^ mbar. PTLs were mounted onto conductive carbon tape and were sufficiently conductive without additional charge neutralization. Spectral processing was performed in CasaXPS software where a Shirley background was applied to all core levels. Pt 4f spectra was fit using asymmetric Lorentzian line shape from literature guidance.^36,37^

#### ToF-SIMS

ToF-SIMS was performed on an TOF-SIMS 5 from IONTOF. Parameters were adjusted depending on the nature of the analysis and the information needed. Positive analysis using MCs^+^ mode was performed with a Bi_3_^+^ primary ion source (LMIG), while negative ion detection used Bi^+^ with an emission current of 0.80 μA. The extraction bias was +25 V (positive mode) and −20 V (negative mode). The secondary ion source for depth profiling varied as well depending on how aggressive the sputter rate needed to be to acquire information in a reasonable time. A low-energy Cs^+^ sputter gun was used for depth profiling in both positive- and negative-ion detection modes. The Cs^+^ acceleration energy was varied from 250 eV (for thin-Pt studies) to 2.0 keV (For Sintered PTLs) with higher acceleration energies enabling access to deeper chemical species or reducing analysis time. For sections where samples were compared over multiple days of analysis, sputter rate was tuned to maintain consistency. Ion beam mixing during sputtering is expected to contribute to some degree of interface broadening; however, because all samples were analyzed within the same material system under identical sputtering conditions, any such effects are consistent across the data set and do not impact the relative interpretation of layer positions or trends. This approach assumes a consistent sputter rate across samples, which is reasonable given the shared material system and identical analysis conditions used to collect the data. IONTOF5 SufaceLab software was used for all postanalysis data processing, including mass spectra, 2D and 3D imaging, and depth profiling analysis. This includes the normalization of intensities to total ion counts as well as erosion rate calculations. Interfaces in the ToF-SIMS depth profiles were identified by averaging the high-intensity regions of the relevant secondary ion signals and defining the interface position as the halfway-intensity point between adjacent layers; this approach provided a reproducible estimate of sputter time across three independent replicates.

In this work, any quantitative comparisons refer to relative changes in normalized ion intensity and sputter time under identical analysis conditions, rather than absolute compositional values (e.g., atomic %), which require system-specific calibration beyond the scope of this study.

MATLAB was also utilized for depth profile plots. In addition to the well-described challenges of surface roughness, shadowing, and porosity, there are difficulties associated with analyzing individual felt fibers, which are relatively small (∼30 μm in diameter) and possess a rounded cross-sectional geometry. These features can exacerbate local angle-of-incidence effects during sputtering and detection, contributing to variable ion yields and depth resolution artifacts that are not always fully mitigated by Region of Interest (ROI) selection or postprocessing. Nonetheless, to reduce mass resolution challenges associated with the complex morphology of PTLs, small analysis areas were isolated. Each sample was analyzed in triplicate within a 50 × 50 μm region centered inside a 250 × 250 μm sputter area, to avoid edge effects and ensure uniform sputtering. This raster size, analysis area, and region of interest were consistently applied to both flat and felt samples. While the ratio between analysis area and raster area differs from standard dynamic SIMS operation, it was intentionally selected to address the significant topographical variation challenges introduced by the felt strands. A larger sputter raster (250 μm^2^) was employed to improve sputtering uniformity across these irregular surfaces and to account for potential analysis drift due to tomography, while a smaller analysis area (50 μm^2^) was chosen to increase ion count density for postanalysis ROI processing. Initial results confirm the reliability of this approach for morphologically challenging samples. Given the varying sizes of individual felt strands, a 14 × 25 μm ROI was consistently selected postacquisition to enable direct comparisons between felt regions.

For the felt PTL samples with varying Pt thicknesses, a secondary sputtering ion energy of 500 eV was used. This setting allowed for the efficient measurement of thicker samples (under 30 min of collection time) while maintaining an acceptable sputter time for the thinnest layers. Notably, for Pt thickness and oxide layer depth profiling, the secondary source was intentionally detuned to 36 nA from the standard 50 nA used with the 500 eV LMIG setting. This adjustment ensured consistent sputter rates over the different days on which data was collected. The selected secondary sputter mode utilized a combined Cs^+^ approach, justified further in the Results and Discussion section.

Proper centering of the X and Y targets for the secondary ion beam on the felt PTL was particularly challenging due to the material’s porosity. To improve alignment confidence, high-ion-count regions, such as overlapping Ti felt areas, were used as reference points. A secondary silicon reference was also employed to verify proper raster centering. Optical imaging during the run and SI imaging postanalysis further confirmed accurate X/Y target positioning. For felt substrates, manual isolation of a clean, relatively flat felt section was necessary. The stage was manually rotated to align the selected felt area parallel to the analysis region, ensuring proper orientation. For sintered materials, selection of a large Ti area with limited pore area was found. For flat substrates, the top-mount stage was used, and no rotation was required due to the absence of porous areas.

## 3 Results and Discussion

### 3.1 Initial Analysis of Pt-Coated Felt PTL

Pt-coated PTL samples were analyzed using both positive (MCs^+^) and negative (Cs^–^) modes; selected ions used to track key species are listed in Table 1. Similarly, CsPt^+^ was chosen for tracking the platinum coating, while Cs_2_O^+^ and Cs_2_TiO^+^ were used to monitor overall oxygen and titanium oxide, respectively. For negative analysis, direct ion species (e.g., O^–^, TiO^–^, PtO^–^) were selected instead of Cs-cluster ions, as they often offer better signal intensity and mass resolution for electronegative species. An example of the ion selection process can be seen in Figure S1.

**Table 1:** Selected Species for PTL Characterization and Their Corresponding Secondary Ions and *m*/*z* Values in Cs^+^ Sputter Depth Profiling, Using MCs^+^ Cluster Ion Detection in Positive Mode (left) and Conventional Negative Ion Detection (right)

	Positive Analysis		Negative Analysis	
Species	Selected Ion	*m*/*z*	Selected Ion	*m*/*z*
Platinum	CsPt^+^	327.87	Pt^–^	197.97
Platinum Oxide	NA	NA	PtO^–^	210.97
Oxygen	Cs_2_O^+^	281.81	O^–^	16.00
Titanium Oxide	Cs_2_TiO^+^	329.89	TiO^–^	63.95
Titanium	CsTi^+^	180.86	Ti^–^	47.95

Figure 2 presents 2D and 3D data from a felt PTL sample with a platinum coating, analyzed using dynamic ToF-SIMS with a Cs^+^ sputtering source. Figure 2a displays individual depth profiles of the selected species, along with overlays to highlight their relative distributions. Depth profiling tracks changes in secondary ion intensity as a function of sputter time, enabling the layer-by-layer chemical analysis of the sample. For the depth profiles in Figure 2a, the intensity was normalized to the total ion count. Data were further selected from the analysis area (50 μm^2^) to a region of interest (14.1 × 25 μm) (for more information see Experimental Section), which allowed consistent isolation of only a single felt of the PTL, reducing noise from topography and morphology.

**Figure 2:**
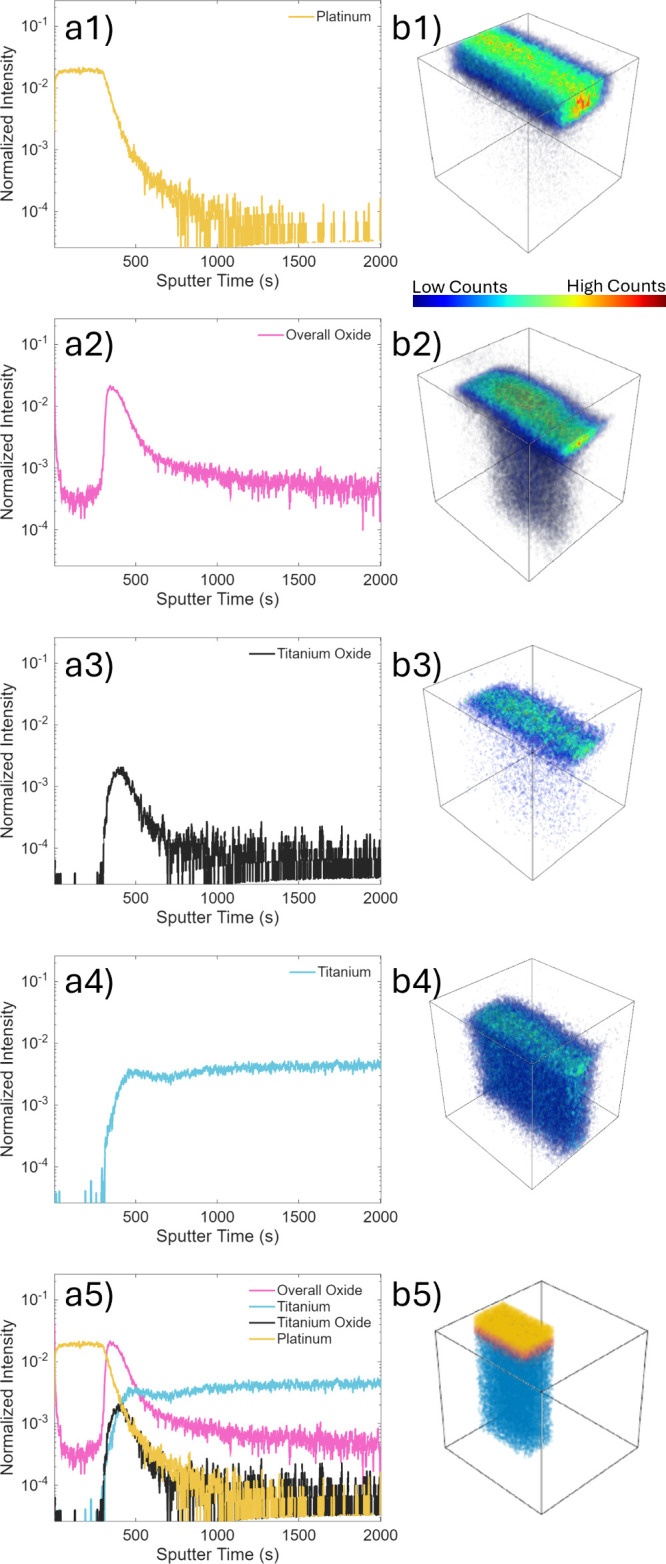
Cs^+^ dynamic ToF-SIMS a) depth profiles and b) 3D reconstructions for 1) Platinum (CsPt^+^), 2) overall oxide (Cs_2_O^+^), 3) titanium oxide (Cs_2_TiO^+^), 4) titanium (CsTi^+^), and 5) all listed species.

Figure 2a1 displays the platinum signal. As expected, the normalized signal intensity is at its highest at the surface of the sample, and plateaus until around 400 s, where the signal begins to decrease. This indicates that the Cs^+^ source has sputtered through the thickness of the platinum coating by this sputter time. Figure 2a2 and 2a3 display the overall oxide and the titanium oxide, respectively. Both signals show a clear intensity increase at ∼400 s, consistent with the presence of a buried interfacial oxide layer beneath the platinum. This is expected, as no oxide removal or reduction treatment was performed prior to deposition, and the oxide likely represents a native titanium oxide layer modified during Pt coating. In addition to the increase at 400 s, there is also a higher oxide signal in the first 50 s of the overall oxide signal (Figure 2a2). This can likely be attributed to transient effects and matrix effects of the surface oxidation of the sample. Figure 2a4 shows the titanium signal, which first increases and then plateaus at ∼500 s, indicating that the Cs^+^ sputter has reached the bulk titanium of the PTL by this time. The overlay of the species described above shown in Figure 2.a5 highlights that there is a clear separation between the platinum coating, the interfacial oxides, and bulk titanium as a function of sputter time.

ToF-SIMS experiments also collect 2D spatially resolved ion images for each scan, providing complementary spatial insight into the average depth profile data. These data can be visualized as either 3D reconstructions or 2D ion images. Figure 2b shows 3D rendering for platinum, overall oxide, titanium oxide, titanium, and an overlay of all of the listed species, respectively. The homogeneity of the platinum coating is more clearly observed in the 3D rendering shown in Figure 2b1, indicating that the coating is well-dispersed on the felt PTL. The 3D reconstructions of the overall oxide and titanium oxide shown in Figure 2b2,b3, respectively, indicate that the oxide layer is also very homogeneous on the felt Ti. Finally, an overlay of all 4 species shown in Figure 2b5 confirms that all species are homogeneous in the *X*–*Y* plane. It is crucial to acknowledge the relative nature of the measurements, as ToF-SIMS cannot independently determine the physical thickness due to matrix-dependent yields. However, in this study, STEM measurements were later used to calibrate erosion rates and estimate the thickness from sputter times. Nevertheless, the overall reconstruction offers a valuable means of visualizing dynamic ToF-SIMS data.

Figure 3 presents the 2D ion images of the relevant species of interest. Figure 3a shows the entirety of the area of analysis, the same area used for the 3D reconstructions in Figure 2b. Figure 3b displays the same data after the region of interest is applied, which relates to the area used for the depth profiles in Figure 2a. This information is similar to that provided in the 3D reconstructions, only condensed into top-down accumulative ion counts. The ion images can also be selected for selected regions in the depth profile instead of the accumulation throughout the run, as shown in Figure 3c-e, which highlights regions discussed previously that isolate ion maps for specific layers within the PTL, the platinum coating (c, 0–50s), the oxide layer (d, 450–500s), and the bulk titanium (e, 1250–1300s). In this way, areas identified by depth profiles for different layers can be visualized spatially in 2D to assess coating homogeneity as well as tracing specific oxides vs overall oxides and pinpointing contamination on the sample. For this example, it can be noted that both the oxides and platinum coating are homogeneously distributed over the region of interest.

**Figure 3:**
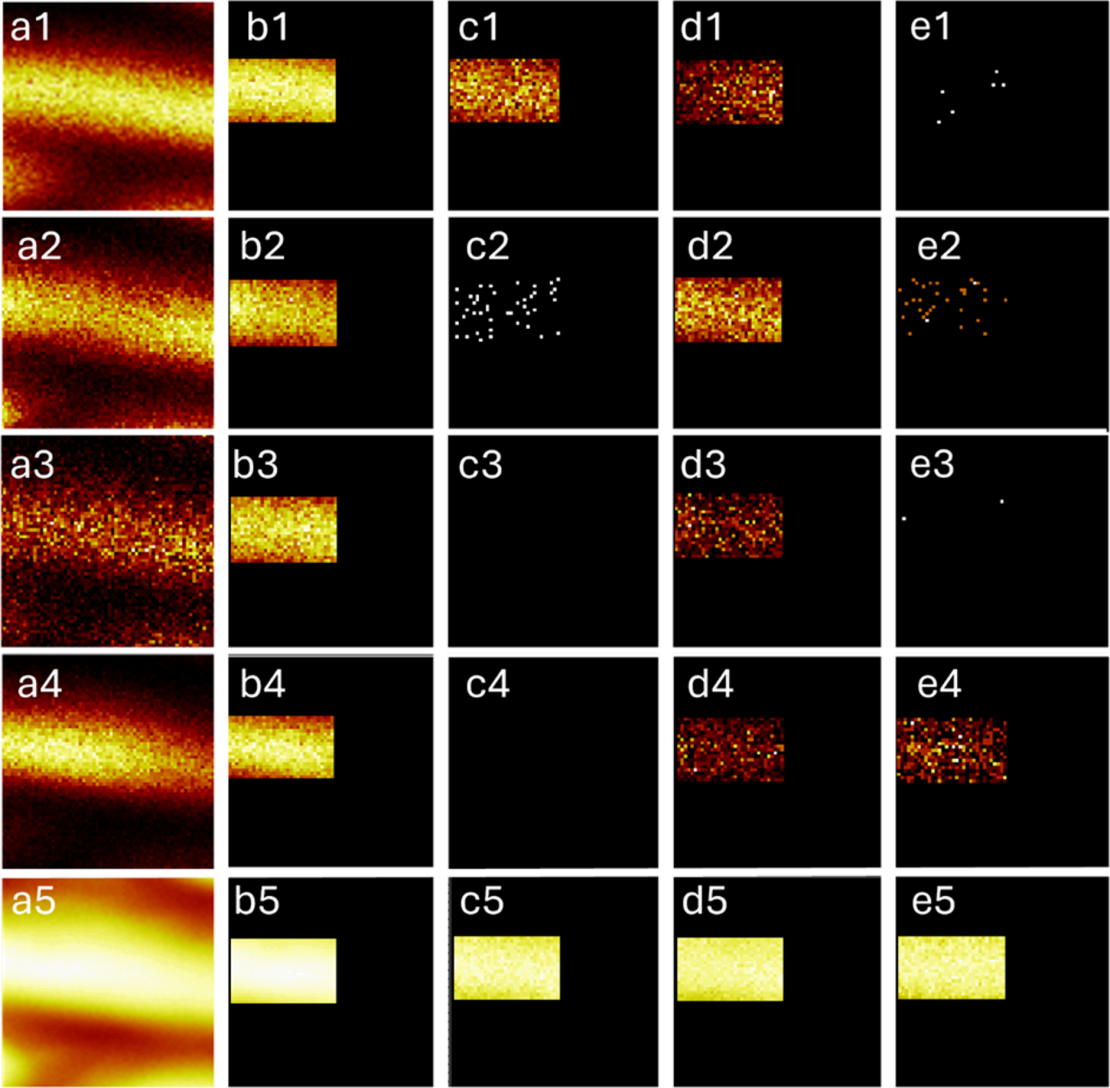
2D ion mapping for a Pt-coated PTL. Rows: 1) platinum (CsPt^+^), 2) overall oxide (Cs_2_O^+^), 3) titanium oxide (Cs_2_TiO^+^), 4) titanium (CsTi^+^), 5) overall ion signal. Columns: a) Total area of analysis total depth accumulation, 50 × 50 μm; b) region of interest as applied to depth profiles, (14.1 × 25 μm) for total depth accumulation; c) region of interest at 0–50 s sputter time; d) region of interest at 400–450 s sputter time; e) region of interest at 1250–1300 s sputter time.

Visualizing the total ions helps to identify regions that may not be ideal for PTL characterization and pinpoint the optimal ROI for the felt PTL isolation. Regions that initially appear ideal may later prove suboptimal due to local tilt (*Z*-axis misalignment), overlapping fibers, or embedded contaminants that mimic the coating morphology. These issues can distort depth profiles and lead to misinterpretation if not identified through spatial imaging. Using a nonideal ROI can lead to false interpretation or inaccurate comparisons of data. Nonideal selected areas are often difficult to identify using a mass spectrum or depth profile alone, highlighting the importance of assessment of 2D ion images during ToF-SIMS experiments or postcollection analysis. Additionally, this suggests that the platinum coating is spatially homogeneous in this area, indicating full coverage of the PTL. The distinct separation between the platinum coating, buried oxide, and bulk titanium demonstrates that when carefully isolated and properly profiled, ToF-SIMS can yield reliable layer separation even in morphologically complex PTLs. The identification of the buried oxide layer is challenging with STEM or STEM-EDS, which is the typical characterization technique used in the field to evaluate the coated PTLs. The combination of ToF-SIMS with STEM-EDS can lead to powerful information about the morphology and chemistry of the protective coated PTL.

### 3.2 Evaluation of Pt-Coated Felt PTLs with a Focus on Pt Coating Thickness Differences

In this section, ToF-SIMS depth profiling is used to evaluate differences in platinum-coating thickness across felt PTLs prepared with varying deposition times. Results from felt PTLs are first compared to flat Ti reference substrates to assess morphological effects on sputtering behavior, followed by correlation with cross-sectional STEM thickness measurements. These samples were prepared by adjusting platinum sputter deposition times to 1, 2.5, 5, and 10 min. Flat substrates coated under identical deposition conditions were analyzed as the reference samples. Depth profiles from felt PTLs and flat substrates are compared to evaluate how surface morphology and porosity influence sputtering behavior during ToF-SIMS analysis and how these differences affect the interpretation of platinum coating thickness.

Figure 4 shows the platinum signal (CsPt^+^) collected using a Cs^+^ sputter source and analyzed in the positive ion mode. Each profile was isolated to a defined region of interest and normalized to the total ion count. While normalization helps reduce overall intensity variation, surface roughness, and porosity in the felt PTLs can still distort the shape of the depth profile. The overlaid traces illustrate how the platinum deposition time influences signal behavior in both flat substrates (Figure 4a) and felt PTLs (Figure 4b). The depth profiles for the flat substrates (Figure 4a) exhibit clear distinctions between deposition times, with the sample coated for 1 min having the shortest sputter etching time, followed by samples having 2.5, 5, and 10 min Pt deposition time, respectively. Because the flat substrates are morphologically uniform, the depth profiles are cleaner and more consistent across all three regions analyzed. Their smooth surfaces promote uniform sputtering and reduce topographical artifacts, allowing for a more reliable comparison between deposition times.

**Figure 4:**
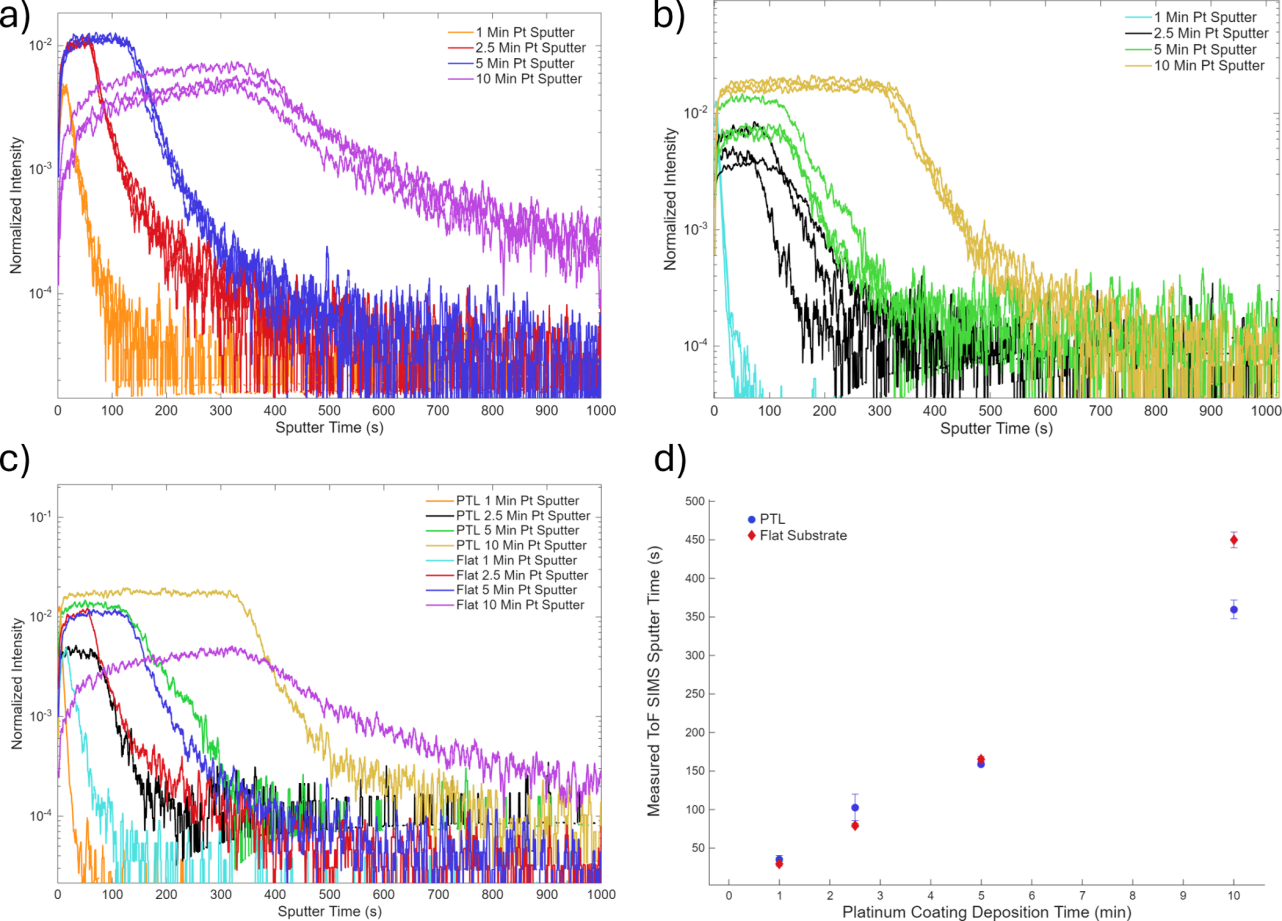
ToF-SIMS depth profiles of the CsPt^+^ signal. a) Pt-coated flat Ti substrates, shown in triplicate. b) Pt-coated felt PTLs, shown in triplicate. c) Selected depth profiles for each sample (flat substrate and PTL) at all Pt deposition times. d) Scatter plot of Pt thickness, represented by the halfway intensity sputter time for all samples.

In contrast, the depth profiles for the felt PTLs (Figure 4b) show greater variability and less sharply defined Pt–Ti interfaces, likely due to local roughness and porosity. This results in signal trailing, where the CsPt^+^ intensity gradually decreases rather than showing a sharp transition. One method to address this is normalizing the CsPt^+^ signal to the Cs^+^ count, which can help account for fluctuations in sputter rate and improve comparability. Extended Dynamic Range (EDR) acquisition is also recommended to ensure accurate capture of both Cs^+^ and Cs_2_^+^ peaks used in normalization. However, as shown in Supplementary Figure S3, normalization to Cs^+^ produced only minor visual differences compared to total ion count normalization, suggesting that trailing in this case is dominated by morphological effects rather than ion yield fluctuations alone. Variability in the CsPt^+^ signal is particularly evident in the 2.5 and 5 min felt PTL samples, where the Pt-to-Ti transition occurs over a broader range of sputter time compared to flat substrates. Despite this variability, averaging the high-intensity regions and calculating the halfway intensity point provides a reproducible estimate of sputter time across three replicates. For the 2.5 min sample, which shows the most variation in Figure 4b, the calculated halfway point is 102.6 ± 17.2 s. Although this standard deviation is larger than that of the 5 min sample (159.0 ± 2.2 s), it is still sufficient to distinguish between different coating thicknesses. Standard deviations for all sputter times are reported in Table S3, illustrating the influence of PTL morphology on depth profiling precision.

To visualize the correlation between ToF-SIMS depth profiling on the flat substrates and felt PTLs, Figure 4c overlays the depth profiles of both sample sets highlighting their respective trends. For this comparison, the felt PTL with the highest total ion count and the visually ‘flattest’ appearance as detected with postanalysis ion imaging was selected. The manual selectivity process emphasizes the challenge of accurately characterizing felt PTLs using ToF-SIMS due to their surface roughness. Consequently, integrating depth profiles, mass spectra, ion imaging, and 3D reconstructions is necessary to identify optimal regions for analysis, as discussed previously. After downselection, the CsPt^+^ signals for the 1, 2.5, and 5 min Pt-coated PTLs closely follow the same sputter time trend as those of the corresponding flat substrates. A significant difference in sputter times for the 10 min platinum deposition is observed, particularly in the normalized ion count (*y*-axis) and the average sputter time for the platinum layer (*x*-axis). While the ion count does not affect the cutoff point of sputering time, the variation in sputter time, especially for the 10 min sample, suggests potential matrix differences. Oxygen is known to promote the formation of CsO-containing clusters (e.g., Cs_2_O^+^). A direct comparison of the oxygen and platinum signals for the 10 min PTL and the 10 min flat substrate is shown in Figure S5, supporting oxygen-related suppression of the CsPt^+^ signal. One possible explanation for the anomalous behavior of the 10 min flat substrate is a higher oxygen-related signal in that sample. Under Cs^+^ sputtering with positive ion MCs^+^ detection, oxygen- or oxide-rich matrices can modify cesium retention, local work function, and the relative yields of oxygen-containing and metal cesium secondary ions. The higher Cs_2_O^+^ response observed for the 10 min flat substrate in Figure S5 is therefore consistent with an oxygen-related matrix effect that contributes to the reduced normalized CsPt^+^ intensity. Because the relative Pt thickness comparison is based on the sputtering time to a consistently defined halfway intensity interface position of the same CsPt^+^ profile, this intensity effect is not expected to dominate the thickness trend. However, matrix-dependent sputter rate differences and profile broadening may still contribute some uncertainty to the apparent interface position.

The sputter times for the flat and PTL samples reported in Table S3 are similar within error. However, the 10 min sample shows a particularly notable deviation, as visualized in the scatter plot in Figure 4d. It remains unclear whether this variation arises from real differences in platinum thickness or from morphological effects such as porosity or surface roughness that impact the sputter rate. One possible explanation is partial platinum loss during deposition on the porous felt surface. Because Pt is deposited via a line-of-sight technique, a fraction of the deposited material can penetrate into or be lost within the pores of the felt rather than form a continuous surface layer, leading to a lower coating thickness.

To validate the platinum thickness trends derived from ToF-SIMS sputter time analysis, cross-sectional STEM measurements were performed on the same set of coated PTLs. STEM imaging of cross sections of the PTL coatings prepared by focused-ion-beam (FIB) was used to assess the accuracy of ToF-SIMS and correlate sputter time with thickness. Figure 5a displays representative high-angle annular dark-field (HAADF)-STEM images along with thickness measurements for the Pt-coated felt PTLs. Figure 5b shows EDS map of the 10 min deposition confirms that the coating is Pt as well as the presence of a native oxide layer (red). Additional data regarding thickness measurements including averages over 5 measurements and standard deviations for each sample is reported in Table S4 (Supporting Information). Significant variation in platinum thickness was observed both across different regions of each sample and between the two sides of individual samples. For the 1 min deposition, the average measured thickness was 6.7 ± 0.76 nm. The 2.5 min sample exhibited notable variability, with “side 1” measuring 18.3 ± 0.48 nm, approximately 4 nm thicker than “side 2,” which measured 13.5 ± 2.0 nm. The 5 min sample showed an average thickness of 31.2 ± 3.4 nm, along with increased variability between regions compared to the 1 and 2.5 min samples. The 10 min sample also displayed side-dependent differences, with side 1 measuring 50.5 ± 3.2 nm and side 2 measuring 55.0 ± 2.3 nm. These results support the conclusion that differences in ToF-SIMS depth profiles between flat substrates and felt PTLs (Figure 4c) reflect real material variation rather than instrumental artifacts. As platinum is deposited via a line-of-sight sputtering technique, thickness heterogeneity is expected. Notably, the range of the Pt coatings explored in this work are lower in thickness than conventional Pt coatings. Although it is not possible to correlate specific ToF-SIMS profiles with “side 1” or “side 2” of each STEM sample, the agreement between techniques suggests that much of the observed ToF-SIMS variability stems from physical differences in the coating rather than limitations in measurement precision. Figure 5b presents the STEM-EDS elemental map of the 10 min sample, where the oxide layer is visualized in red and subsequently measured as 7 nm. While this technique provides reliable measurements of oxide layer thickness, it offers only elemental information, so specific oxide speciation cannot be directly identified, though it may be inferred.

**Figure 5:**
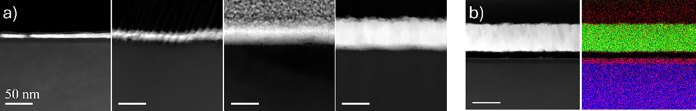
HAADF-STEM analysis of Pt-coated PTLs with Pt deposited for: a) 1, 2.5, 5, and 10 min deposition. b) STEM-EDS overlay map of Pt-coated PTL 10 min; Pt (green), Oxygen (red), Titanium (blue). All scale bars are 50 nm.

Following STEM platinum layer thickness measurements, ToF-SIMS depth profiles were converted from sputter time to calculated thickness (nm) based on erosion rates, where the average thickness measured by STEM for side 1 of the 10 min platinum sample (55 nm) was used to calculate the erosion rate. These calculations are reported in Table S4. Based on the halfway peak intensity of the Pt signal, thicknesses were calculated for the other samples, along with their standard deviations. Figure 6 compares the platinum thicknesses derived from ToF-SIMS and STEM measurements across different deposition times. Although only the 10 min STEM measurement was used for the erosion rate conversion, the calculated thicknesses from ToF-SIMS closely match those obtained from STEM for all investigated samples. The consistency of these results demonstrates ToF-SIMS’s ability to estimate Pt thickness across various samples with reasonable accuracy using a single known reference thickness. The similar standard deviations observed in both methods further suggest that the variability in platinum thickness arises from the material itself, introduced during deposition, rather than from limitations in the ToF-SIMS technique. This highlights ToF-SIMS as a rapid and effective tool for estimating platinum thickness variations, particularly in post-testing scenarios where thickness changes are critical.

**Figure 6:**
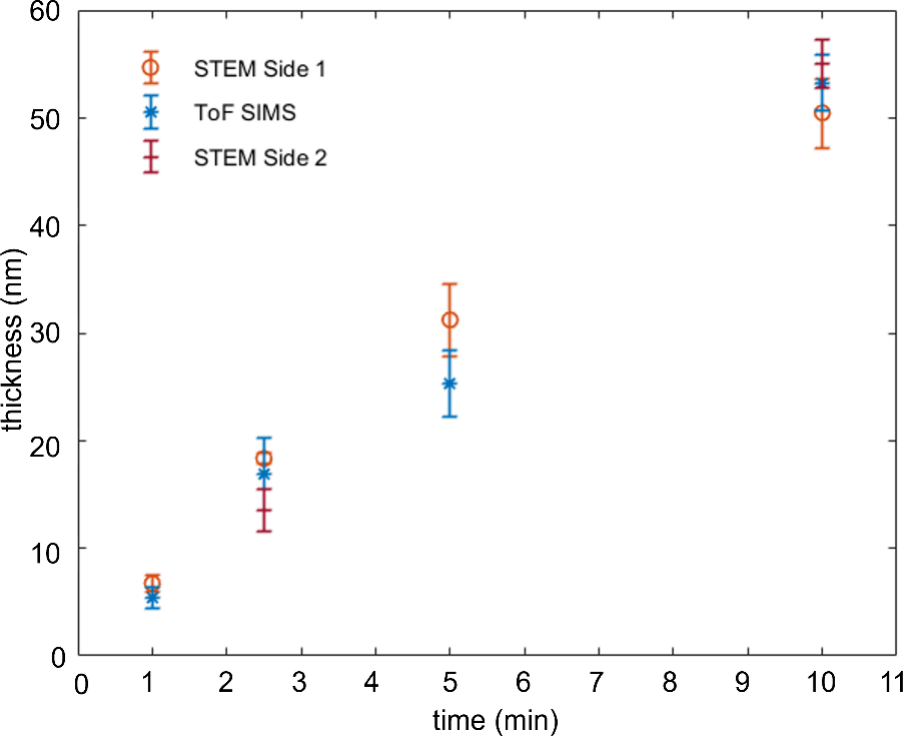
Pt thickness measurements obtained from STEM imaging with thickness values derived from ToF-SIMS sputter time conversion for PTLs coated with Pt for 1, 2.5, 5, and 10 min as a function of Pt deposition time (minute) and coating thickness (nm).

### 3.3 Evaluation of Pt-Coated Felt PTLs with a Focus on Buried Oxide Interface

Building on the established sensitivity of ToF-SIMS to platinum coating thickness, this section focuses on characterization of the buried oxide interface beneath the Pt layer. Depth profiles collected in both positive and negative ion modes are used to assess oxide layer consistency and composition across samples. Figure 7 presents depth profiles for the same samples reported in the previous section but now displaying the titanium oxide signal and overall oxide signal, to highlight the interfacial oxide layer. Figure 7a shows the repeatability of the interfacial oxide profile across three areas of the 5 min felt PTL. Repeatability is assessed by comparing the width of the oxide-related signal in sputter time, with peak centers occurring around 200 s. The thickness of the oxide layer and titanium oxide layer is consistent for all 3 areas. A subtle variation is observed in the titanium oxide signal (purple), but the width of the layer remains consistent, as indicated by the uniform peak profile. The overall oxide signal follows a similar trend to the titanium oxide signal but displays greater variability in intensity beyond 400 s, suggesting localized differences in the surrounding matrix or oxide composition between analyzed regions. If the matrix conditions were fully consistent across all areas, the overall oxide signal would be expected to follow the same trend as the titanium oxide signal, with a peak centered at approximately 200 s. It is expected that there would be a consistent buried oxide layer here as no treatment to remove the native oxide layer was conducted before platinum deposition.

**Figure 7:**
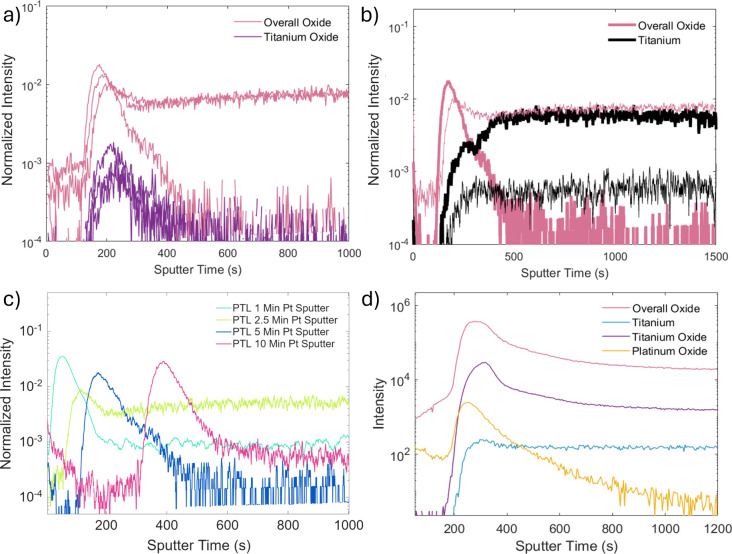
a-c: Cs^+^ depth profiles: a) three areas on the 5 min Felt PTL: overall oxide and titanium oxide signal; b) two areas on the 5 min Felt PTL: overall oxide and titanium signal; c) one area each of the 1, 2.5, 5, and 10 min PTL, overall oxide signal; and d) Cs– depth profile.

The variation in the overall oxide intensity is further examined in Figure 7b. Figure 7b highlights two different areas of the 5 min felt PTL. Each profile shows the overall oxide signal as well as the bulk titanium signal. One area exhibits a suppressed overall oxide signal, whereas the other shows an enhanced oxide signal. To distinguish the 2 areas, area 1 is plotted with a thicker line than area 2. Notably, one of the areas has a higher overall oxide concentration in comparison with the normalized total ion count. This can be explained in a similar argument given in the suppression of the platinum signal for the 10 min flat substrate signal. As the depth profiles expressed are normalized to the total ion count, the signal differences may arise from local matrix effects, where an increase in the titanium signal can suppress the apparent oxide intensity after 400 s. In the case of the enhanced oxide signal (thinner line), the correlated titanium signal is lower, suggesting an overall change in the bulk matrix of the sample. In comparison, the thicker line (area 1) with a suppressed overall oxide signal has a stronger titanium signal within the bulk of the sample.

Figure 7c shows the overall oxide species for the 1, 2.5, 5, and 10 min felt PTL samples. Visually, the thicknesses of the oxide layers are relatively consistent between the samples. Within the conditions investigated, the buried oxide layer thickness appears to be independent of platinum deposition time. The uncoated PTL can be seen in Figure S6, which shows a constant decrease in the total oxide from the surface through the titanium bulk material, further confirmation that this oxide layer is native to the PTL surface. This characterization establishes a baseline for comparing future samples, including those treated to reduce surface oxide or those subjected to degradation, where the buried oxide layer may have grown.

While Cs^+^ mode using MCs^+^ cluster ion detection offers many advantages for depth profiling, it has reduced sensitivity for species that typically form negative secondary ions, such as many oxides. Although acceptable for high-concentration species, the detection of oxide species like platinum oxides, which are present at low concentrations in the PTL matrix, is particularly challenging. Figure 7d shows an example of depth profile in Cs^–^ mode, highlighting the distribution of the overall oxide vs the distribution of oxides associated only with Pt (PtO^−^) or Ti (TiO^−^). With all three oxides, an increase in intensity is observed after the surface but before the appearance of titanium signal. This is followed by a decrease in intensity when the titanium signal arises. The overall oxide signal can be visually assessed as a combination of signals from platinum oxides and titanium oxides. The PtO^–^ signal peaks earlier in sputter time than the TiO_2_^–^ signal. This analysis with Cs in negative mode indicates that platinum oxide exists in this matrix; however, its signal intensity is too low to be detected using the MCs^+^ mode. It is important to note that in Cs^–^ mode, the Pt^–^ signal is saturated due to its high intensity, making it difficult to analyze accurately. As a result, Pt^–^ is not included in Figure 7d. Instead, the presence of platinum is confirmed using the Cs^+^ mode, where the signal is more suitable for interpretation. This highlights the importance of conducting two sets of experiments, one with positive and one with negative mode. The negative mode is expected to be particularly informative for the characterization of samples subjected to modifications to alter the interfacial oxide or to monitor the evolution of oxide after degradation studies. These findings establish a consistent buried oxide baseline that is largely independent of platinum deposition time, providing a reference for future studies targeting oxide modification or degradation.

### 3.4 Analysis of PTLs with Thin Pt Coating

This section demonstrates the sensitivity of ToF-SIMS for detecting ultrathin platinum coatings and trace interfacial species that are challenging to resolve by using conventional microscopy techniques. A major advantage of ToF-SIMS is its exceptional sensitivity to detect extremely low concentrations, making it especially useful when other analytical techniques lack the necessary sensitivity. Figure 8a presents the ToF-SIMS positive depth profile, and Figure 8c presents 3D reconstructions of selected species for a felt PTL coated with a thin layer of platinum. ToF-SIMS successfully identified the presence of platinum and oxygen at the PTL surface. Figure 8b presents the negative depth profile for the felt PTL along with 3D reconstructions of selected species of interest. Here, platinum, platinum oxide, titanium, titanium oxide, and overall oxide distributions are highlighted. This provides a clearer view of platinum oxide, which appears at low intensity in the MCs^+^ depth profile (Figure 8a) but is more evident in the negative-ion mode.

**Figure 8:**
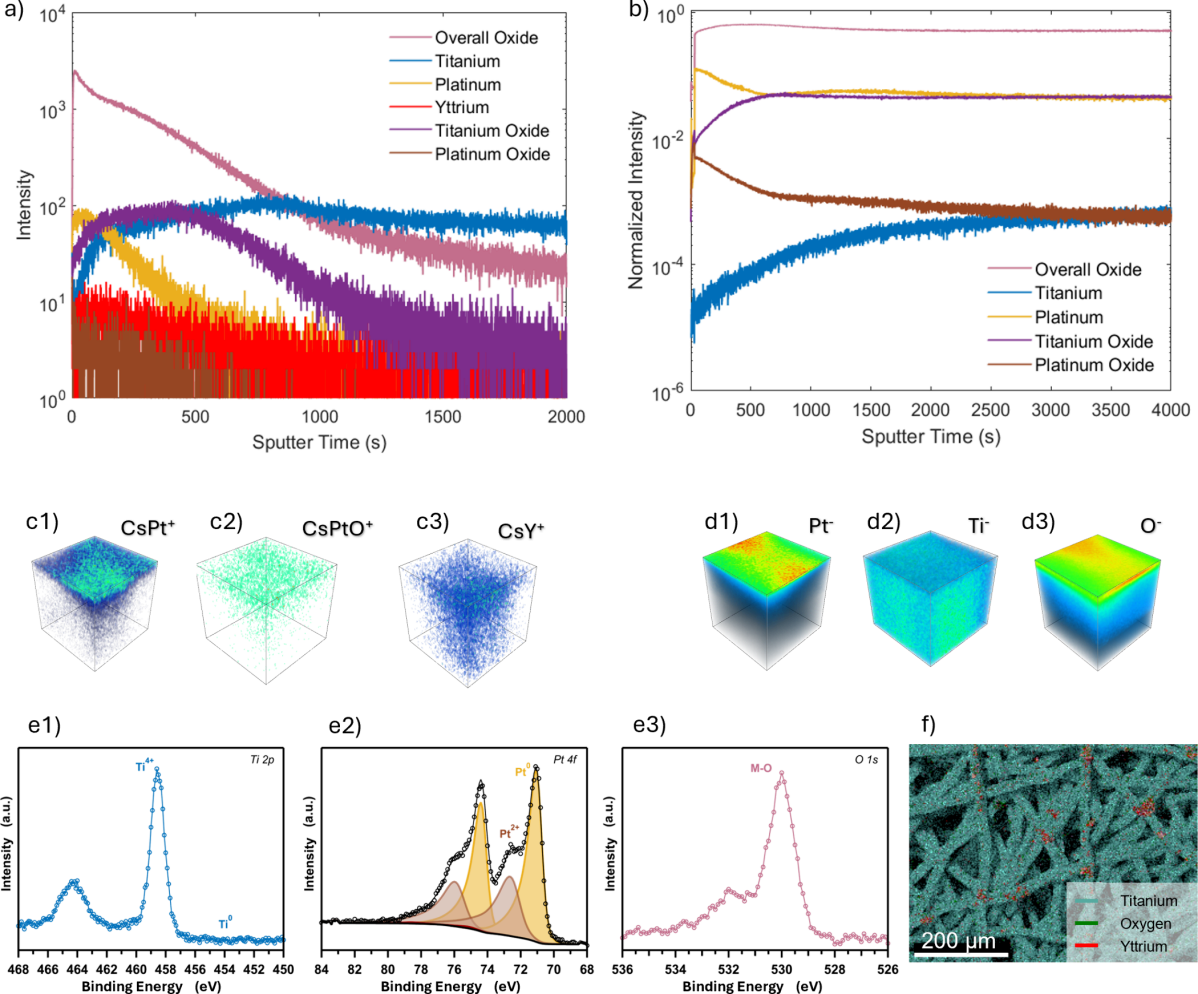
Felt PTL with thin Pt coating: a) ToF-SIMS Cs^+^ depth profile; b) ToF-SIMS Cs– depth profile and 3D reconstructions for selected species of interest; c-d) ToF-SIMS 3D reconstructions: c1) CsPt^+^, c2) CsPtO^+^, c3) CsY^+^, d1) Pt^–^, d2) Ti^–^, d3) O^–^; e) XPS peaks: e1) Titanium 4f, e2) Platinum 4f, and e3) Oxygen 1s; and f) EDS map.

The platinum and platinum oxide exist on the surface of the material, and the titanium oxide is buried below the platinum and platinum oxide at the titanium interface. 3D reconstructions indicate that platinum forms a well-dispersed, homogeneous thin coating (Figure 8d1), while the overall oxide signal arises from overlapping contributions of platinum oxide and titanium oxide species.

To further validate these findings, XPS analysis was conducted on the same PTL. Figure 8e shows the high resolution Pt 4f, Ti 2p, and O 1s spectra. The Pt 4f spectrum confirms the presence of the Pt on the surface. Both Pt(0) and Pt­(II) are detected, indicating that the coating contains both metallic Pt and PtO. Titanium­(IV) is the predominant peak in the Ti 2p spectrum. This indicates that there is titanium oxide (TiO_2_) present within the detection range of the XPS (∼2–10 nm), further indicating that the platinum layer is very thin or not homogeneously dispersed on the felt Ti PTL. Finally, the O 1s range shows a metal-oxide bond, verifying the presence of metal oxides at the surface of the felt PTL. While it is not possible to differentiate whether the entire surface of platinum coating is oxidized, or oxide is located at the interface with Ti, XPS is sensitive enough to confirm the presence of both types of species. SEM-EDS (Figure 8f) analysis revealed the presence of yttrium in these samples, appearing as agglomerates on certain felt regions and depicted in red in the EDS map. During ToF-SIMS experiment, these areas with yttrium agglomerates were specifically avoided. However, even the areas with no visible yttrium contamination revealed the presence of yttrium signal at the titanium interface, demonstrating high sensitivity for trace contamination. Moreover, the 3D reconstructions indicate that yttrium is more concentrated in localized areas, confirming its presence as a localized contamination. Notably, yttrium is positioned between the Pt and Ti layers. The spatial distribution of yttrium is consistent with contamination introduced prior to platinum deposition, likely originating from the sintering process.^33^ This exemplifies the value of ToF-SIMS in contamination analysis, where mass spectra can be searched for unexpected elements.

This case study highlights the exceptional sensitivity of ToF-SIMS for identifying ultrathin coatings and trace interfacial species that are not readily detected by SEM-EDS. By integration of depth profiling with mass spectrometry and 3D visualization, it provided a comprehensive understanding of material composition, confirming the expected and detecting unexpected elements.

### 3.5 Visualization of Sintered PTLs with 3D Renderings

Sintered PTLs are also of interest and are often investigated as the base material for porous transport electrodes (PTEs) alongside felt PTLs. However, their complex morphology and larger required analysis areas present additional challenges compared with felt PTLs. Figure 9 presents 3D rendered images of a Pt-coated sintered Ti PTL, collected in positive mode over a 70 × 70 μm analysis area. The platinum signal was used to assess coating uniformity across the larger area. In the analyzed regions, the platinum signal indicates uniform coating coverage across the entire area. The titanium oxide follows the shape of the underlying titanium, but the overall oxide distribution suggests a surface oxide contaminant. Additionally, silicon contamination was detected beneath the platinum layer, indicating that this contamination was present before the Pt deposition process.

**Figure 9:**
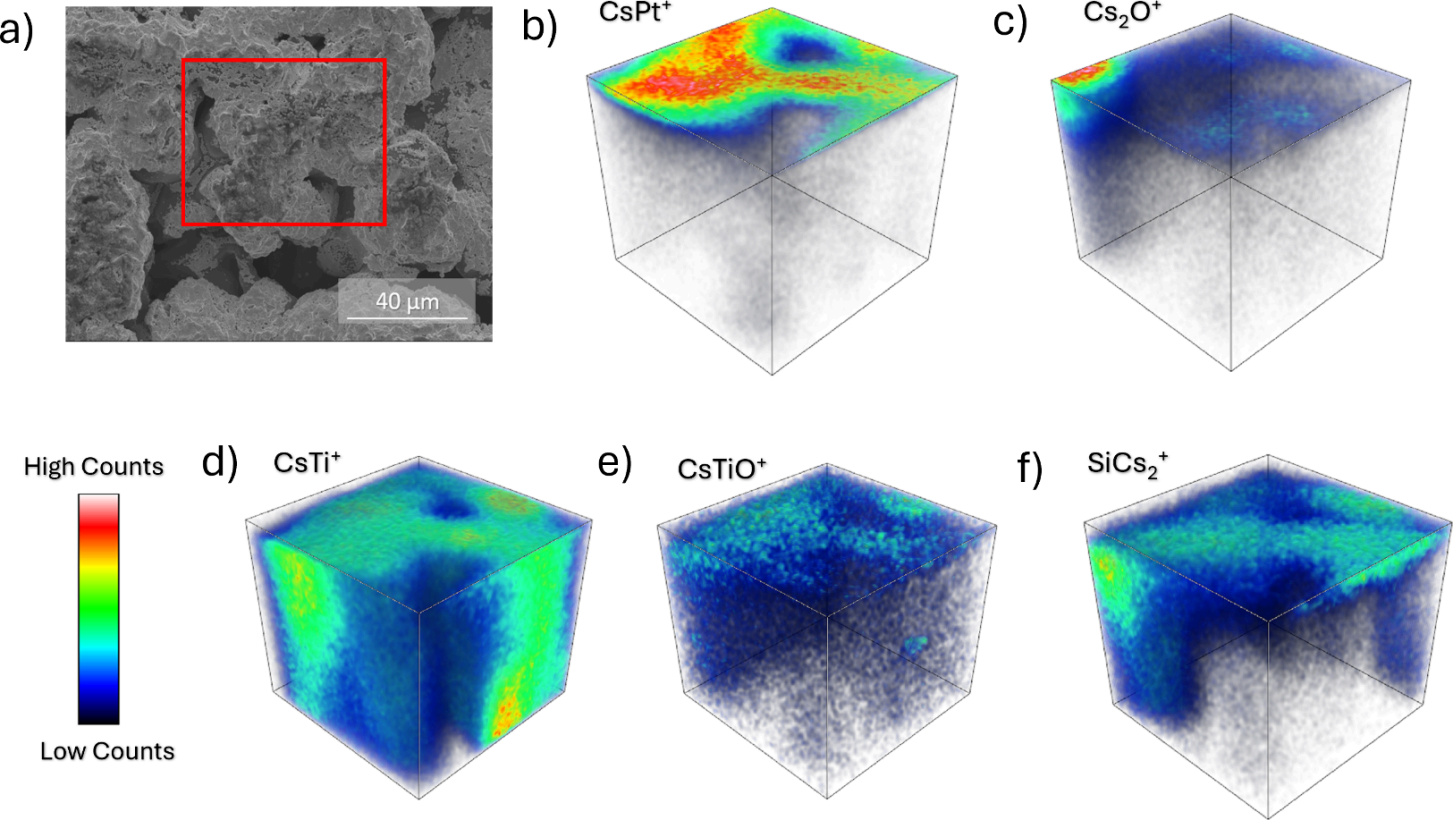
ToF-SIMS analysis of a sintered Ti PTL with Pt coating. a) SEM-EDS image outlining an example of the analysis area, 70 μm^2^. b-f) 3D reconstruction of 70 μm^2^ area: CsPt^+^, Cs_2_O^+^ CsTi^+^, CsTiO^+^, and SiCs_2_^+^, respectively.

The 3D reconstructions in Figure 9 demonstrate that ToF-SIMS can be used qualitatively for large-area analysis, providing valuable insights into the spatial distribution of the PTL components in three dimensions. This case also further exemplifies the power of ToF-SIMS in identifying unknown contaminants. The ability to detect both known and unexpected contaminants allows for more informed correlations between the material characteristics and performance testing. The analysis of sintered titanium PTLs provides a foundation for characterizing more complex components such as porous transport electrodes, which incorporate catalyst layers into similarly intricate morphologies.

## 4 Conclusion

This systematic study investigated the ability of ToF-SIMS to probe differences in platinum-coating thickness, identify interfacial oxide layers, detect surface contaminations, and investigate larger areas of PTLs. ToF-SIMS depth profiling revealed reproducible tracking of differences in platinum layer thicknesses across Pt-coated felt PTLs ranging from 6 to 55 nm, with results validated through flat Ti reference substrates and STEM-based thickness measurements. While this proof-of-principle study demonstrates the feasibility of correlating ToF-SIMS sputter time with PTL thickness, future studies aiming for quantitative thickness determination are encouraged to employ multiple reference thickness measurements across the analyzed range, as this approach will reduce error propagation and improve calibration accuracy.

The technique further enabled identification of buried oxide layers, with overall oxide and titanium oxide signals detected beneath the platinum coating using both Cs^+^ and Cs^–^ depth profiling modes. In addition, platinum oxide was detected at the interface between platinum and titanium oxide using Cs^–^ profiling, which enhanced ionization of oxide species. For ultrathin coatings, ToF-SIMS detected platinum and platinum oxide signals undetectable by SEM-EDS. XPS confirmed the surface chemistry but lacked spatial resolution, an important limitation addressed by ToF-SIMS through complementary depth-resolved analysis. The use of 2D ion imaging and 3D reconstructions confirmed the spatial homogeneity of surface coatings and buried species. Finally, large area analysis and 3D reconstructions enabled qualitative evaluation of sintered PTLs.

This work established ToF-SIMS as a characterization method for titanium-based PTLs in PEMWEs, addressing long-standing challenges in resolving different layers and, especially, buried interfaces in coated PTLs. By establishing a repeatable and adaptable approach for ToF-SIMS analysis of coated PTLs, this work provides an essential foundation for spatially resolved chemical characterization of Pt-coated PTLs. Collectively, these findings support broader implementation of ToF-SIMS for interfacial analysis in electrochemical systems while outlining key methodological considerations for accurate application to spatially complex materials, including rough, porous, and chemically heterogeneous surfaces. Future studies will extend this technique to the analysis of degraded samples, where ToF-SIMS could provide mechanistic insight into the chemical evolution of platinum coatings and buried oxide layers under realistic operating conditions. The methodology developed in this study can be directly transferred to the investigation of other advanced electrode structures. It can be applied to porous transport electrodes, where the integration of a catalyst layer directly onto the coated PTL introduces additional complexity and challenges for the characterization. This approach is also relevant to the investigation of components used in liquid alkaline and anion exchange membrane electrolyzers, where similar characterization challenges exist.

## Supplementary Material



## Notes

Additional experimental data, including ToF-SIMS depth profiles, sputter time measurements, and STEM thickness measurements (PDF)
