# Supplementary material for: Time of Flight Secondary Ion Mass Spectrometry for Characterization of Pt-Coated Porous Transport Layers in PEM Water Electrolyzers
Source: ACS Appl Nano Mater. 2026 May 25;9(22):10224–37. doi: 10.1021/acsanm.6c00919 (PMC13247959; doi:10.1021/acsanm.6c00919)
Supplement: Supplementary file 1 [file an6c00919_si_001.pdf]

Supporting Information

Time of Flight Secondary Ion Mass Spectrometry for Characterization of Pt-Coated Porous Transport Layers in PEM Water Electrolyzers

Genevieve Stelmacovich [a], J. David Arregui-Mena [b], Michael Walker [a], Jayson Foster [a], Samantha Ware [c], James L. Young [c], Guido Bender [c], Adam Paxson [d], David A. Cullen [e], and Svitlana Pylypenko [a,c,]*

**Corresponding Author. E-mail: spylypen@mines.edu*

a: Department of Chemistry, Colorado School of Mines, Golden, Colorado 80401, United States

b: Materials Science and Technology Division, Oak Ridge National Laboratory, Oak Ridge, TN, 37831, USA

c: S. Ware, J.L.Young, G. Bender: Chemistry and Nanoscience Center, National Laboratory of the Rockies, 15013 Denver West Parkway, Golden, Colorado 80401, United States

d: A. Paxson: Plug Power, Latham, New York 12110, United States

e: D.A. Cullen: Center for Nanophase Materials Sciences, Oak Ridge National Laboratory, Oak Ridge, TN, 37831, USA

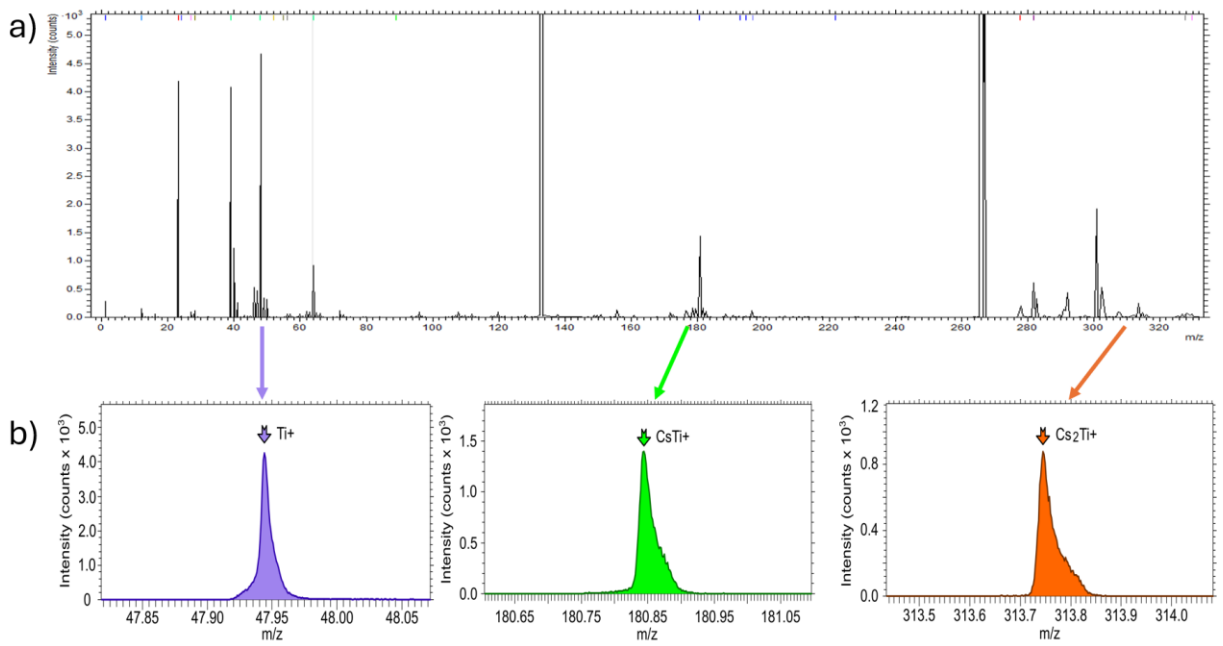

Figure S1. a) Selected range of m/z on Cs^+ depth profile ToF-SIMS spectrum and b) Relevant titanium signals within the selected m/z range.

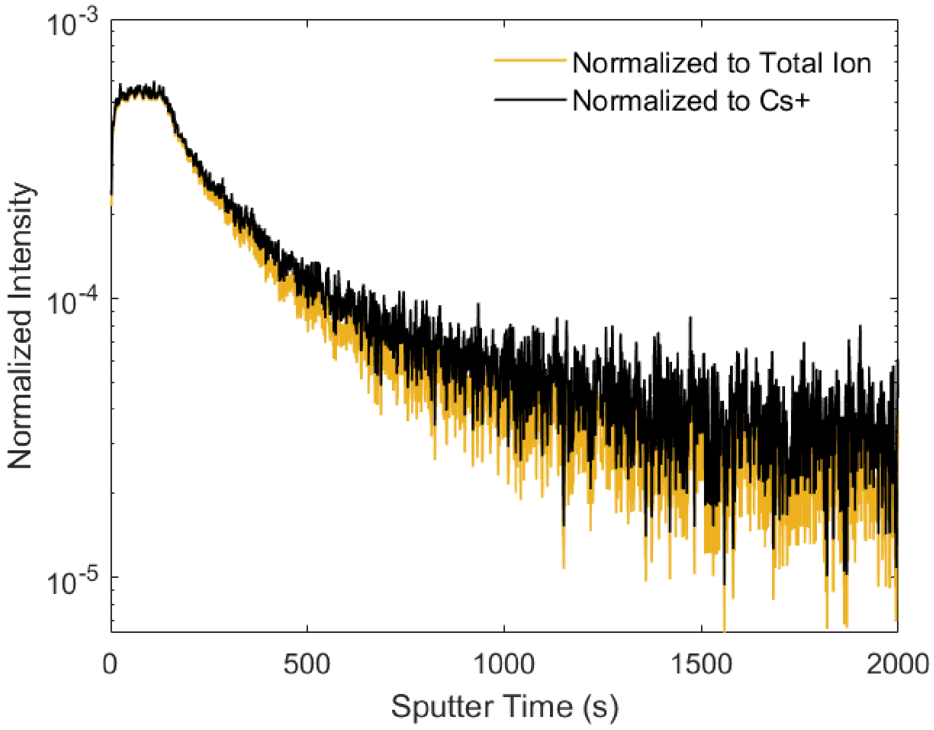

Figure S2. $CsPt^+$ signal of felt PTL normalized to Cs^+ signal rather than total ion count, to account for signal tracing.

Table S3. ToF SIMS depth profiling showing the sputter times at which the $CsPt^+$ signal decreases to 50% of its maximum intensity, used for depth calibration of felt PTLs and flat substrates.

	Felt PTL		Flat Substrate	
Pt Deposition Time (min)	Average Sputter Time (s)	Standard Deviation	Average Sputter Time (s)	Standard Deviation
1	35.2	5.4	29.6	3.2
2.5	102.6	17.2	79.4	2.7
5	159.0	2.2	165.5	3.5
10	359.7	12.2	450.1	10.3

Table S4. Pt thickness measurements obtained from STEM imaging with thickness values derived from ToF SIMS sputter time conversion for PTLs coated with Pt for 1, 2.5, 5, and 10 minutes. ToF SIMS calculated thicknesses, converted from halfway intensity points of $CsPt^+$ signal based on IonTof5 SurfaceAnalysis erosion rate calculations.

	STEM Measurements		ToF SIMS Calculation	
Pt Deposition Time (min)	Average Measured Thickness (nm)	Standard Deviation	Average Converted Thickness (nm)	Standard Deviation
1	6.7	0.76	5.5	1.0
2.5 (Side 1)	18.3	0.48	16.9	3.3
2.5 (Side 2)	13.5	2.0	---	---
5	31.2	3.4	25.3	3.1
10 (Side 1)	50.5	3.2	---	---
10 (Side 2)	55.0	2.3	53.3	2.6

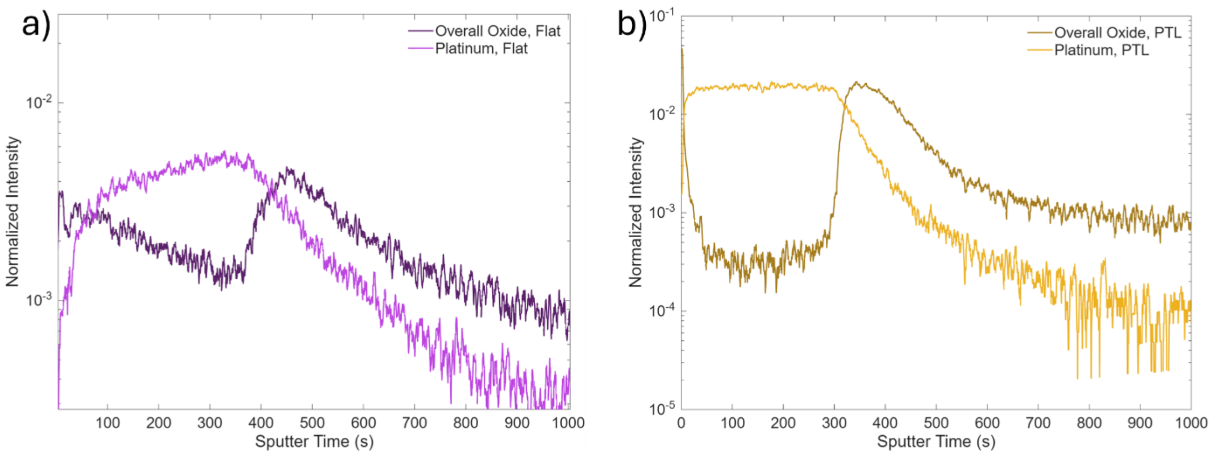

Figure S5: Depth profile of 10-minute sputter, flat substrate and PTL.

a) Flat substrate (Cs+ mode), overall oxide and platinum signal.

a) PTL (Cs+ mode), overall oxide and platinum signal.

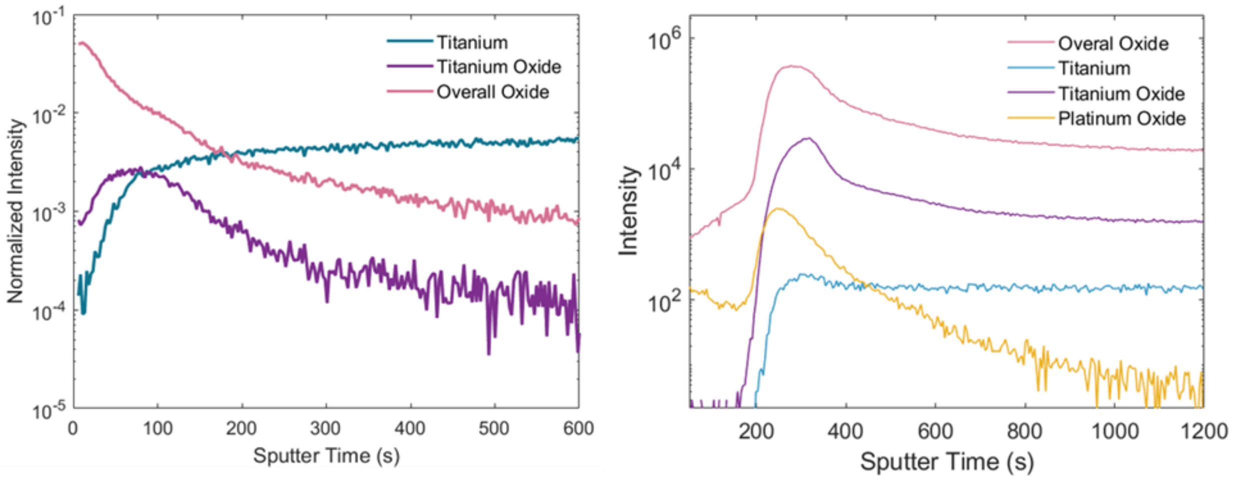

Figure S6: Depth profile of uncoated PTL vs coated PTL.

Left: uncoated PTL (Cs- mode), overall oxide, titanium oxide, and titanium signal.

Right: coated PTL (Cs- mode), overall oxide, platinum oxide, titanium oxide, and titanium signal
